# Development and validation of cost-effective multi-sample hypoxia chambers for proton ultra-high dose rate organoid irradiations

**DOI:** 10.1016/j.ctro.2025.100970

**Published:** 2025-05-01

**Authors:** Rutger Jan Cornelis de Koster, Jeremy Putra Gunawan, Hans Peters, Johan Bussink, Lara Barazzuol, Marc-Jan van Goethem, Alexander Gerbershagen, Robert Paul Coppes, Stefan Both

**Affiliations:** a Particle Therapy Research Center (PARTREC), University Medical Center Groningen, University of Groningen, The Netherlands; b Department of Radiation Oncology, University Medical Center Groningen, University of Groningen, PO Box 30001, 9700 RB Groningen, The Netherlands; c Department of Biomedical Sciences, University Medical Center Groningen, University of Groningen, PO Box 30001, 9700 RB Groningen, The Netherlands; d Department of Radiation Oncology, Radboud University Medical Center Nijmegen, PO Box 9101, 6500 HB Nijmegen, The Netherlands

**Keywords:** Hypoxia, Organoids, Ultra-high dose rate

## Abstract

The tissue sparing of ultra-high dose rate irradiation has been partially observed *in vitro* while controlling oxygen levels. Here, we developed a hypoxia system for high-throughput irradiations that accommodates embedded 3D cultures, such as organoids and spheroids, enabling the study of the effect of oxygen on radiation responses.

## Highlights

• Developed cost-effective, multi-sample hypoxia chambers for 3D culture irradiation.
• Validated chamber design with oxygen measurements using sensors and staining.
• Demonstrated successful oxygen modulation in ECM-embedded 3D cultures.

## Short introduction

The response of cells to ionizing radiation is strongly influenced by oxygen levels, a phenomenon known as the oxygen effect [[1], [2], [3]]. Due to chaotic vasculature and rapid proliferation, tumours often harbour hypoxic tumour areas, leading to radiation resistance. As a result, modifying tumour oxygen levels is an important area of research to improve radiotherapy treatment outcomes [[4], [5], [6]]. Over the years, many studies have investigated the effects of oxygen *in vitro* [[7], [8], [9], [10], [11]]. More recently, the observation that Ultra-High Dose Rate (UHDR) irradiation can spare normal tissue while maintaining tumour control *in vivo* relative to conventional dose rates [12,13] has renewed interest in controlling oxygen levels *in vitro* for radiobiological studies.

Despite the growing interest, most oxygen-controlled *in vitro* studies still rely on 2D conventional culture systems [[14], [15], [16], [17], [18], [19]]. In these experiments, oxygen levels were controlled by using a hypoxia workstation [[14], [15], [16]], hypoxia chamber with mylar foil setup [17,18] or other in-house designs [19]. However, currently available commercial and in-house hypoxia chamber designs are expensive and can only accommodate a limited number of samples [20], limiting the experimental throughput. Additionally, these setups frequently lack validation of actual cellular oxygen concentrations, limiting experimental reliability and throughput.

Advancements in 3D culture models have made them the favoured *in vitro* model. They better mimic physiological conditions by supporting realistic cell–cell interactions, cellular morphologies and tissue-like organisation, leading to better prediction outcomes than monolayer 2D cell cultures [21]. Specifically, organoids are 3D cultures generated from stem cells deriving multiple cell types within a single structure, closely resembling its source tissue, such as mammary gland organoids [22,23]. Patient-derived normal and tumour organoids offer even greater predictive potential by capturing biological and physical mechanisms related to oxygenation and radiation, including the UHDR stem cell sparing hypothesis.

Hypoxia chambers, used to seal cell cultures to prevent experimental interference of the atmospheric oxygen pressure, are indispensable components in the *in vitro* radiobiology research field [18,20]. However, current hypoxia chamber designs are often expensive and can only accommodate one to three samples [20], limiting the experimental throughput. Also, the accuracy of the cellular oxygen concentrations is usually unvalidated and may not take the complexities of oxygen dynamics of 3D cultures into account. Here, we present the development and validation of a simple, cost-effective, multi-sample hypoxia chamber designed to irradiate 3D culture models for radiobiology research applications (such as UHDR research).

## Materials and methods

### Hypoxia chamber design and use description

**Fig. 1:**
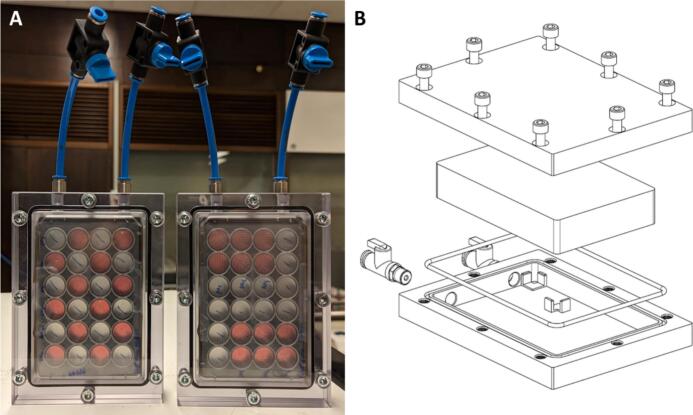
Images of the newly designed and validated hypoxia chambers. **A**: two chambers containing Lumox 24 well plates in which ECM gels containing spheroids are seeded. The chamber on the left is prepared in a checkerboard pattern for scattered beam irradiations. The right chamber is prepared for scanned field irradiations. **B**: An exploded view of the chamber design. For simplicity, the shut-off valves are combined with the inlet and outlet ports, but the final design separates the two components for more flexibility.

The hypoxia chamber (Fig. 1) comprises two 162.0 mm by 120.0 mm, 15.0 mm thick polycarbonate (PC) halves. Both halves (base and lid) are CNC milled to be combined and create a 132.0 mm by 89.6 mm by 26.0 mm centre cavity, dimensioned to fit most standard cell culture/well plates with some margin to allow for gas flow, resulting in 2.0 mm thick PC irradiation windows. The chamber base features a slot around the plate cavity for a No. 162 Viton O-ring (The Chemours Company, DE, USA), enabling an airtight seal. The chamber base and lid are combined using eight M6 screws and helicoils in the chamber base, located away from the chamber cavity, to prevent dosimetric interference and radioactive activation of the metal. The top of the base features two push-in tube connectors type QS-G1/8–6-l (Festo AG & Co. KG, Germany) with o-rings to allow gas flushing of the chamber cavity. Both connectors feature type HE-2-QS-6 shut-off valves (Festo AG & Co. KG, Germany) enabling air-tight transportation of the chambers, e.g. from the culture lab to the experimental room.

To enable oxygen diffusion between the cell cultures and the sealed chamber cavity, Lumox multiwell 24-well cell culture plates (Sarstedt AG & Co. KG, Germany) are used, as the Lumox foil base is gas permeable. The Lumox plates are fixed in place and raised 1.0 mm to create an air gap using four specially designed PC corner pieces to improve air diffusivity in the foil base. To achieve the desired hypoxia level in the cell cultures, the hypoxia chambers should be flushed for 48 h using certified premixed gas mixtures (SOL S.p.A., Italy) of 0, 1, 2 or 5 % O_2_ + 5 % CO_2_ in N_2_ before irradiation. We selected a range of oxygen levels between 0–5 % oxygen as it encompasses the range found in physiology. Typically, 21 % oxygen, as in air at normal atmospheric pressure, is known as normoxia [24]. A range of 4–7.5 % of oxygen is expected in peripheral tissue, known as physioxia [24]. Oxygen concentrations below 2 % are usually regarded as hypoxia [24]. The chambers should be placed in an incubator at 37 °C to facilitate cell growth before (during flushing) and after irradiation.

Whereas conventional monolayer cell cultures are seeded directly on the bottom surface of each well, 3D cultures cells are embedded in an extracellular matrix (ECM) gel to accommodate 3D growth and better mimic the physiology of tissues.

### Details for proton irradiation setup design

The chambers are designed to be irradiated horizontally or vertically (horizontal beamlines). For the latter, the wells need to be completely filled with growth media and sealed using parafilm (Amcor, USA). The plate wells can be irradiated individually or with a larger field. For individual irradiation of the 24 plate wells, a 20.0 mm by 20.0 mm square collimator should be used for scattered proton beams. In this case, at most, twelve wells in a checkerboard pattern should be used (Fig. 1A, left chamber plating scheme) to prevent significant scatter dose contributions to neighbouring wells. This method of irradiation is required for UHDR irradiations with scatter beams. For larger, more homogenous fields, e.g. Pencil Beam Scanning, wells can be grouped closer together (Fig. 1A, right chamber). Depending on the field size, two groups of up to eight wells can be irradiated within one chamber without significant scatter contributions from two separate irradiations. For this use case, it is advised to keep two empty wells between groups (e.g. Fig. 1A, right chamber plating scheme).

For organoid irradiations, it should be noted that the ECM gel is less than 5.0 mm thick. Therefore, the dosimetry procedure for shoot-through irradiations can be executed simply by determining the output factor using a dosimeter at the intended sample location along the beam axis.

### Chamber gas tightness confirmation

To validate the gas tightness of the chamber design, an EZO-O_2_ oxygen sensor (Atlas Scientific, NY, USA) was modified to be battery-powered and fit entirely in the chamber cavity. After starting the sensor and sealing it in the chamber, the chamber was flushed to 0.0 % while placed in an incubator at 37 °C.

### Physical and biological oxygen level validation

The ECM gel used with organoids introduces a barrier for oxygen diffusion [25]. This warrants extensive validations of the matrix O_2_ concentration. Therefore, we performed a validation on the physical and biological levels.

For both validations, spheroids were generated by seeding MDA-MB-231 cells in suspension with the extracellular matrix, reduced growth factor basement membrane extract (Cultrex, USA) at a 1:2 ratio. Upon gelation, growth media consisting of DMEM (Gibco, USA) supplemented with 10 % FBS (Biosera, France) and 1 % Penicillin-Streptomycin (Invitrogen, USA) is added to accommodate cell growth.

**Fig. 2:**
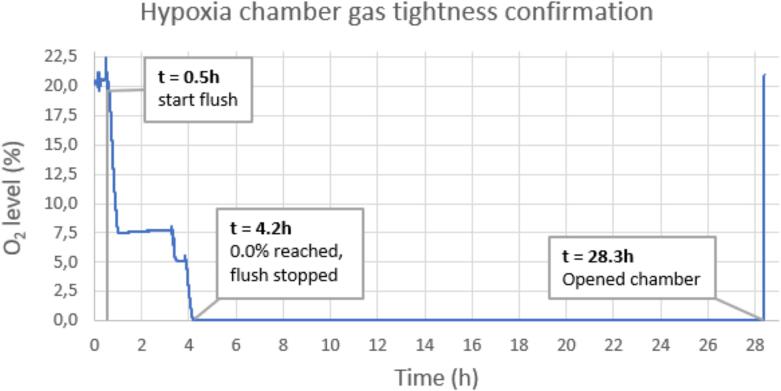
Oxygen level in a sealed hypoxia chamber placed in a 37 °C incubator, recorded for > 24 h using an EZO-O_2_ sensor. The chamber was flushed with 0 % O_2_ and 5 % CO_2_ in N_2_ gas at a very low flow rate. The recorded steps in the oxygen level decrease are due to flushing pauses meant to test if specific oxygen levels could be reached. At t = 4.2 h, 0.0 % O2 was reached, and flushing was stopped. After 24 h at 0.0 % O2, the chamber was opened and confirmed airtight.

First, a physical oxygen validation was performed using a needle-type fibre-optic NTH-PSt7 oxygen microsensor in combination with a Microx 4 oxygen meter (PreSens Precision Sensing GmbH, Germany). One chamber lid was modified to contain 24 holes, sealed with foam septa to allow the fibre-optic microsensor to access the ECM gel and media for oxygen concentration measurements while preventing the reintroduction of atmospheric oxygen (Fig. 2A). This measurement was replicated independently at least twice for each respective gas mixture.

Second, a biological oxygen validation was performed using the Hypoxyprobe-1 kit (Hypoxyprobe, USA). The Hypoxyprobe-1 kit uses pimonidazole hydrochloride, which readily forms stable covalent adducts with thiol groups in proteins, peptides and amino acids below 5–10 mmHg O2. These adducts can then be detected by a monoclonal antibody, visualising hypoxia in cells and tissues [26]. The spheroids are collected by opening the chambers and rapidly dissolving the ECM gel with 37 °C dispase, centrifuged, fixed in 4 % Formaldehyde, and stained with the hypoxyprobe-1 kit according to the manufacturer’s instruction.

## Results

### Chamber gas tightness results

Using the EZO-O_2_ oxygen sensor, the gas tightness of the chamber design was confirmed to last for 24 h, without detecting any oxygen level increase (Fig. 2). The chamber was reopened after the 0.0 % O_2_ reading passed the 24 h mark.

### Physical oxygen level validation

**Fig. 3:**
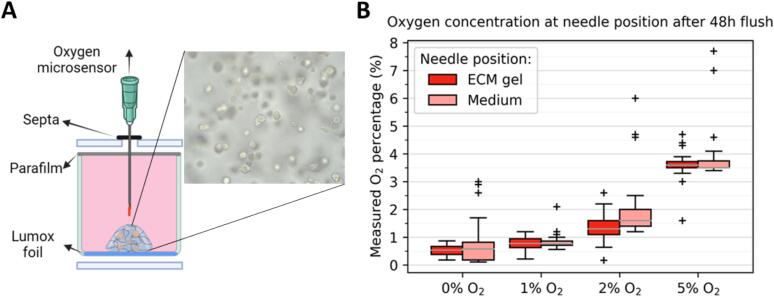
Designed hypoxia chamber accomplishes hypoxic levels in 3D cultures. **A.** Schematic overview of physical oxygen measurement in basement membrane extract extracellular matrix (ECM) gel containing MDA-MB-231 spheroids and media in a Lumox well. Created in Biorender. **B.** Physical oxygen concentration measurement by the PreSens oxygen sensor system. The boxes represent the following means and standard deviations (whiskers); in basement membrane extract ECM gel, 0 % O_2_ = 0.52 ± 0.19 % [N = 37, three experimental replicates], 1 % O_2_ = 0.76 ± 0.24 % [N = 23, two replicates], 2 % O_2_ = 1.3 ± 0.50 % [N = 25, two replicates] and 5 % O_2_ = 3.6 ± 0.54 % [N = 24, two replicates]. In medium, 0 % O_2_ = 0.72 ± 0.73 % [N = 37, three replicates], 1 % O_2_ = 0.85 ± 0.30 % [N = 23, two replicates], 2 % O_2_ = 2.2 ± 1.2 % [N = 25, two replicates] and 5 % O_2_ = 3.9 ± 1.1 % [N = 24, two replicates].

To physically validate the oxygen at the cell level in the Matrigel/BME (ECM), we inserted the Presens oxygen microsensor as close as possible to the cells (Fig. 3A). The physical oxygen validation results are provided in Fig. 3B. The ECM gel measurements showed an increase with increased O_2_ flushing for 48 h (Fig. 3B).

### Biological oxygen level validation

**Fig. 4:**
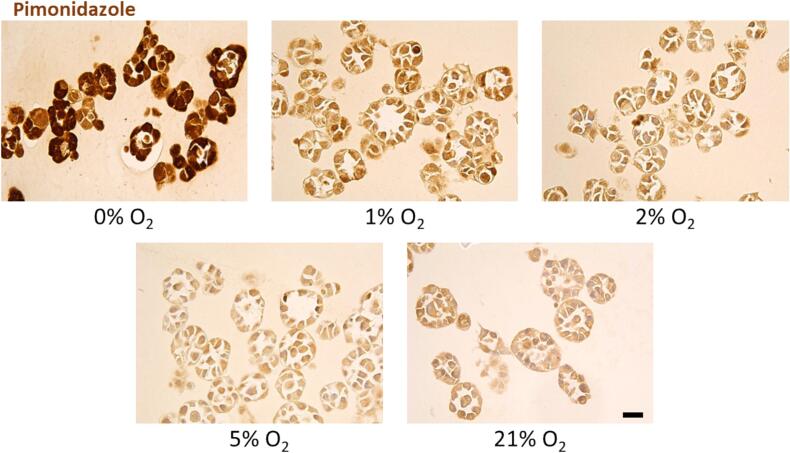
Visualization of hypoxia in MDA-MB-231 spheroids after 48 h of flushing using the Hypoxyprobe-1 kit. Scale bar represents 25 µm. A prominent brown staining is observed in the spheroids flushed with 0 % and 1 % O2 relative to 21 % O2. The staining difference between 2 %, 5 % and 21 % O2 is less prominent, in agreement with the sensitivity of the pimonidazole.

Next, MDA-MB-231 spheroids were collected, fixed and stained using the Hypoxyprobe-1 kit to show biological hypoxia following gas flushing (Fig. 4.). Through the staining, a prominent positive brown staining is observed in spheroids flushed with 0 % and 1 % oxygen relative to 21 % oxygen. Meanwhile, the staining differences between 2 %, 5 % and 21 % oxygen gasses were less prominent, in agreement with the absence of bioreduction at higher pO_2_ levels, i.e., 5–10 mmHg corresponding to approximately 1 % O_2_.

## Discussion

In this study, we aimed to create a flexible system to control oxygen levels during irradiation of embedded 3D cultures which allows for multiple samples. Here, we proposed a hypoxia chamber design that enables modulation of the oxygen level in ECM-embedded 3D cultures. Crucially, embedded 3D cultures are better at mimicking tissue and tumour physiology than conventional monolayer cultures, as they allow for better cell–cell and cell-ECM interactions [23,27]. These chambers can be used to study the radiobiological effect of hypoxia and physioxia in spheroids and organoids.

Physical validation of the hypoxia chamber revealed that after 48 h of flushing, the control of oxygenation of the culture is established, both within the ECM and the growth media. Interestingly, after gassing with 1–5 % oxygen, we found that the measured oxygen levels in the ECM and media were lower than the oxygen concentration of the supplied gasses (Fig. 2). This may have been caused by the oxygen consumption of the spheroids within the culture also in-line with presently available literature [14,26,28]. Thus, stressing the importance of oxygen concentration validations of various in-house hypoxia systems used in many groups to improve experimental replicability.

In cells, changes in molecular oxygen readily induce various adaptive mechanisms [29]. Molecular oxygen levels were monitored by oxygen sensors, which are transiently stabilised during hypoxia and rapidly degraded as oxygen concentration increases. For instance, HIF-1α, a well-known transcription factor responding to oxygen level, is degraded with a half-life of 5–8 min [30]. A more extended time period was required to retrieve our spheroids from our hypoxia culture system, so a more stable hypoxyprobe-1 kit was used to visualise hypoxia. However, the threshold for pimonidazole staining is below ≤ 10 mmHg oxygen, robustly detectable in only the 0 % and 1 % oxygen flushing conditions (Fig. 3) [31,32]. Notably, as our spheroids are smaller we achieved an uniform hypoxia, while other work has shown a gradient of oxygenation between the core and extremities of a larger spheroids. [14,33].

A few limitations remain to our design. First, the extracellular matrix required for the 3D culture presents a barrier to oxygen diffusivity [25]. Therefore, it is necessary to conduct 48 h of gassing to prepare the samples for irradiation to ensure the oxygen levels have reached equilibrium. Second, there is a necessity to design and use new, specially manufactured corner pieces when using well plates shaped differently than the Lumox plates in this work. These corner pieces, however, do enable flexibility in choosing the types of plates to be used. Third, while the cultures are in the hypoxia chamber, there is no way to manipulate the culture without opening the hypoxia chamber which may present as a limitation in some experiment designs. Fourth, some time is required to open the chamber which may prevent experimental investigation of hypoxia-sensitive phenotypes that degrade rapidly, such as HIF-1α. To address the final two limitations, the chambers could be used together with a hypoxia workstation.

In summary, we have created a cost-effective, multi-sample hypoxia chamber design that supports embedded 3D cultures to allow radiobiological research, simulating physioxic and hypoxic conditions needed for the mechanistic studies, e.g. on the effects of UHDR irradiations. The files for replicating the hypoxia chamber design can be provided by the corresponding author upon request.

## Funding information

This collaboration project is co-financed by the Dutch Ministry of Economic Affairs and Climate Policy by means of the PPP-allowance made available by the Top Sector Life Sciences & Health to stimulate public-private partnerships (reference PPP-2021-27); Ion Beam Applications SA (IBA Ltd), Louvain-la-Neuve, Belgium; University Medical Center Groningen (UMCG), Groningen; and the Cock-Hadders fund (reference 2024-70).

## Declaration of competing interest

The authors declare that they have no known competing financial interests or personal relationships that could have appeared to influence the work reported in this paper.
